# Undiagnosed Osteoporotic Vertebral Fractures in an Octogenarian During the Coronavirus Disease Pandemic

**DOI:** 10.7759/cureus.38585

**Published:** 2023-05-05

**Authors:** Gabriel Siu Nam Ng, Linda Yin-king Lee, Eric Chun-Pu Chu

**Affiliations:** 1 Chiropractic and Physiotherapy Center, New York Medical Group, Hong Kong, CHN; 2 School of Nursing and Health Studies, Hong Kong Metropolitan University, Kowloon, HKG

**Keywords:** octogenarian, pathological fracture, pandemic, chiropractor, chiropractic, diagnosis, covid-19, elderly, vertebral fractures, osteoporosis

## Abstract

Osteoporotic vertebral fractures are frequently misdiagnosed or under-recognized in the older population, leading to disease progression and reduced quality of life. This case of an 87-year-old woman with acute back pain highlights the importance of early diagnosis and management of fragility fractures. During the coronavirus disease (COVID-19) pandemic, patients with a history of well-managed osteoporosis experienced worsening symptoms of vertebral collapse due to activity limitations and prolonged immobilization. The initial diagnosis of spinal stenosis delayed appropriate treatment for four months. Serial magnetic resonance imaging revealed compression fractures at L1 and L3, and a dual-energy x-ray absorptiometry scan showed osteoporosis with a T-score of −3.2. Pharmacological therapy, including bisphosphonates, was initiated. A comprehensive rehabilitation program with a multidisciplinary approach, with bracing, and lifestyle changes helped stabilize the spine, reduce pain, and maximized function. Her condition improved with close monitoring and guidance during home exercises. This case exemplifies the necessity of a precise and timely diagnosis of osteoporotic vertebral fractures to initiate management and mitigate disease progression.

## Introduction

Osteoporosis and subsequent pathological fractures are under-recognized causes of morbidity and mortality in the older population [1]. Distinguishing between normal age-related changes and pathological processes is increasingly challenging [2]. Concomitant medical conditions and atypical presentations of the disease can obscure an accurate diagnosis and delay appropriate treatment. Here, we present the case of an 87-year-old woman with a history of long-term osteoporosis management who suffered pathological vertebral compression fractures that were initially misdiagnosed, highlighting the importance of accurate diagnosis and management of the fractures in geriatric patients with multiple comorbidities.

Osteoporosis is a systemic skeletal disorder characterized by decreased bone mass and microarchitectural deterioration of the bone tissue with a consequent increase in bone fragility and susceptibility to fracture [3]. Alendronate, a bisphosphonate that inhibits bone resorption, is the first-line treatment for osteoporosis [4]. Although long-term alendronate use has been shown to increase bone mineral density and decrease fracture risk, inadequate treatment or intermittent use may not fully mitigate fracture risk, especially in older patients with other risk factors, such as cancer treatment, glucocorticoid use, pharmacological therapy with teriparatide or bisphosphonates, or malnutrition [5].

Pathological vertebral compression fractures caused by osteoporosis can lead to severe pain, functional impairment, decreased pulmonary function, and diminished quality of life [6]. Despite the significant individual and public health impacts of osteoporotic vertebral fractures, they remain underdiagnosed, partly because of the attribution of symptoms to more benign age-related processes [2]. Chiropractors undergo extensive education and training to become experts in the spine and nervous system [7,8] and utilize clinical guidelines for the management of postmenopausal osteoporosis [9]. While severe pathological conditions causing low back pain are not commonly seen in chiropractic clinics [10], chiropractors are experienced in effectively managing pain with minimal side effects [11]. They are well-versed in diagnosing the cause of back pain and providing preventative and drug-free treatment options [12]. In this case study, we aimed to increase clinical suspicion of pathological vertebral fractures, especially in high-risk geriatric patients with a history of long-term osteoporosis, to avoid dangerous diagnostic delays. 

## Case presentation

An 87-year-old woman presented to a chiropractor with a two-year history of worsening lower back pain. The pain began gradually without injury and was initially intermittent but remained constant over the past six months, during which time she had weakness in the bilateral lower extremities and decreased mobility. The pain was rated 7/10 in severity, which worsened with activity and limited her ability to perform the majority of the activities of daily living independently. Her quality of life was scored at 56% on the World Health Organization Quality of Life questionnaire. Lying in a supine position provided her relief. Her medical history included osteoporosis, which had been well managed with alendronate for 13 years. She had also undergone a thyroidectomy 10 years prior and had stable cardiovascular disease. Her last bone density test and spinal radiographs showed that her osteoporosis had remained stable; however, she developed lumbar degenerative joint disease before the coronavirus disease (COVID-19) pandemic. During the COVID-19 pandemic, her osteoporosis medication (alendronate) was not taken constantly for three years. Eight months before presenting to a chiropractor, she had been hospitalized for COVID-19 pneumonia for two weeks. She reported an unintentional five-pound weight loss over the past six months due to poor appetite, and her back pain and symptoms had worsened significantly since hospitalization. She was treated with a muscle relaxant (cyclobenzaprine), pain medication (paracetamol), and a nonsteroidal anti-inflammatory drug (etoricoxib) for degenerative joint disease.

**Figure 1:**
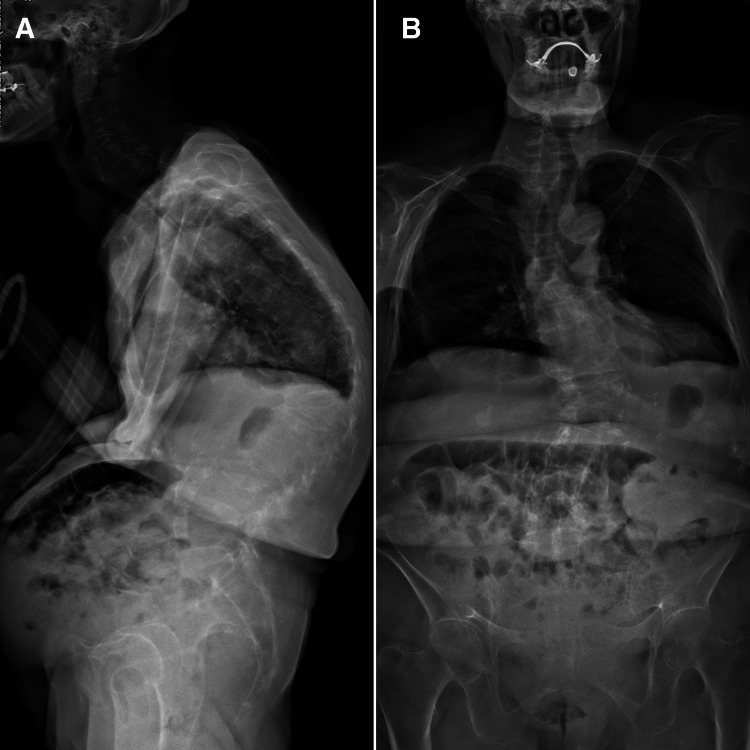
Full-spine radiograph. (A) Generalized decreased bone density is indicative of osteopenia/osteoporosis and renders assessment suboptimal at the frontal view. Degenerative changes with marginal osteophytes in the thoracic and lumbar spine. Compression fracture from T11-L1 with marked loss of vertebral height. Mild loss of vertebral height is seen in the L3 vertebral body. Collapse of T9 was also seen. These are likely osteoporotic in nature. (B) Moderate thoracolumbar scoliosis with convexity to the left. Some of the vertebral bodies cannot be well assessed on the lateral view.

**Figure 2:**
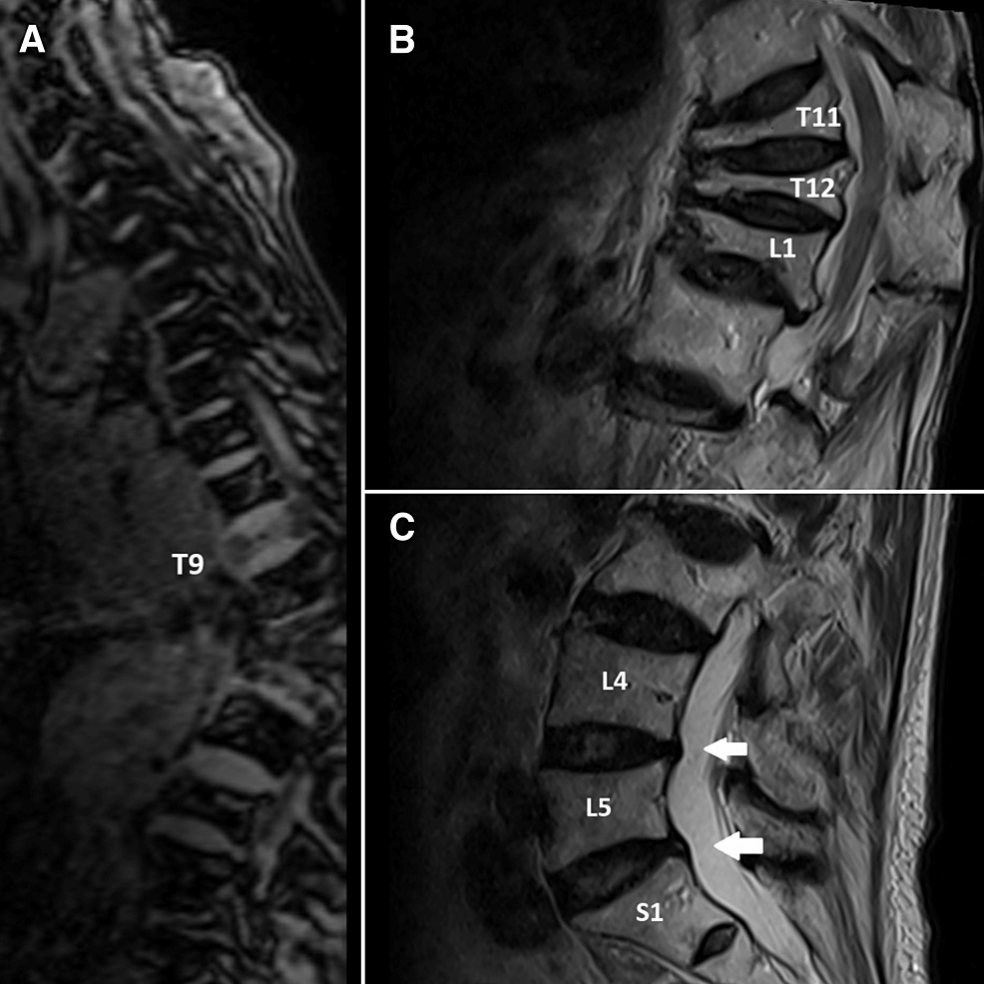
Magnetic resonance lumbar spine. (A) Collapse of T9 also seen in localizer images, which implied osteoporotic in nature. (B) Compression fracture from T11, T12, and L1 with marked loss of vertebral height. These are likely osteoporotic in nature. (C) The lumbar discs are normal in height. Mild loss of vertebral height is seen in the L3 vertebral body. At L4/5 and L5/S1 levels, mild posterior disc bulge and facet joint degeneration causing bilateral lateral recess stenosis.

The chiropractor's examination revealed that the patient had a hyperkyphotic posture and an antalgic gait and required minimal assistance to get on and off the exam table. The lumbar range of motion was moderately reduced in all planes due to the pain. Tenderness to palpation was marked at the T12-L1, L1-2, L4-5, and L5-S1 vertebral levels. Straight leg raise testing was negative for radiculopathy but reproduced her leg pain when the legs reached 60° bilaterally. Muscle strength was 4/5 in bilateral hip flexion and full strength in the lower extremities. Reflexes were normal. Full-spine radiographs taken by the chiropractor showed multiple compression fractures with osteophytes in the thoracic and lumbar spine, vertebral wedging at the T11-L1 levels, and general osteopenia of the skeletal system (Figure 1). Magnetic resonance imaging revealed loss of vertebral height at T11, T12, L1, and L3 and moderate thoracolumbar scoliosis with minimal cord compression (Figure 2). A dual-energy X-ray absorptiometry scan was then arranged, which showed osteoporosis with a T-score of −3.2. Based on the patient’s history, clinical examinations, and imaging results, an acute exacerbation of chronic low back pain due to osteoporotic vertebral compression fractures at T11-T12, L1, and L3 with associated muscle spasming was diagnosed. Underlying degenerative changes also contributed to the overall disability in the setting of long-standing osteoporosis.

The multidisciplinary approach plan was focused on providing symptomatic relief, stabilizing the spine, and preventing future fractures. As there is the potential harm of high-velocity spinal manipulations on fragile bones, all forceful manual techniques were avoided. The treatment included gentle active joint movement to the mid- and lower thoracic and lumbar spine and avoiding the fracture sites, instrument-assisted soft tissue mobilization to the paraspinal muscles, isometric training by exercise machine (AllCore360°, Georgia, USA) to strengthen core muscles, therapeutic ultrasound three times per week to reduce inflammation, and a walking regimen beginning at 5-10 min per day and increasing as tolerated to promote spinal stabilization and conditioning. A custom rigid thoracic-lumbar-sacral orthosis brace was also provided to support the fracture site and reduce movement when out of the office. Weight-bearing precautions, proper lifting and bending techniques, vitamin D/calcium repletion, dietary recommendations, and bone health education were provided to prevent future injuries. The patient was then referred to an endocrinologist from the same medical group for additional osteoporosis medications and monitoring. An orthopedic surgeon kept track of the fracture status while in-house physiotherapists kept track of a personalized rehabilitation program and fall prevention techniques.

She demonstrated moderate improvement over 12 visits, with pain reduction from 7/10 to 2/10 intensity, improved mobility and range of motion (ability to walk a few blocks), and reported a return of appetite and weight gain (four ponds). Her quality of life score had improved to 78%. Her outcomes remained limited by her underlying condition; however, symptom stabilization and limited functional return were considered reasonable therapeutic goals. With telemedicine, home health resources, and continued rehabilitative care, future exacerbations or fractures can still be avoided as osteoporosis advances. 

## Discussion

The older population has been disproportionately affected by COVID-19, with a higher risk of developing severe and critical cases that often require hospitalization [13]. Particularly, older adults infected with COVID-19 face an increased likelihood of acute respiratory distress syndrome (ARDS), an inflammatory condition of the lungs [13]. The excessive production of proinflammatory cytokines observed in COVID-19 and prolonged inactivity during hospitalization may accelerate bone loss and breakdown, especially in aging and vulnerable individuals [13]. Although glucocorticoids are commonly administered to reduce harmful inflammation and support respiratory function in patients with ARDS, they also pose the risk of bone loss when used long-term or at higher doses. In older patients hospitalized with COVID-19, glucocorticoid therapy for severe respiratory illnesses can cause bone loss associated with inactivity and increase the risk of developing secondary osteoporosis [13].

A delay in the diagnosis of osteoporotic vertebral fractures during the COVID-19 pandemic can culminate in the exacerbation of symptoms and advancement of the disease due to immobilization and limited mobility [14]. This case highlights the critical importance of precise diagnosis and consideration of osteoporotic pathological fractures, which can manifest clinically in a manner similar to spinal stenosis and osteoarthritis [2]. Pathological vertebral fractures secondary to osteoporosis are common causes of back pain in the older and need to be differentiated from spinal stenosis or osteoarthritis [2], which was the incorrect initial diagnosis in this case. During this period of the pandemic, older individuals were often confined to their homes or isolated in hospitals with restricted activity to curb viral transmission, which affected the diagnosis and management of osteoporosis [14]. Prolonged rest and inactivity cause rapid bone loss and increase the risk of fragility fractures in osteoporotic individuals [15]. Accurate diagnosis of acute osteoporotic vertebral fractures is essential to institute appropriate treatment and management to provide pain relief, stabilize the spine, prevent future fractures, and optimize mobility and quality of life, especially in the geriatric population [16]. A delay in diagnosis, as evidenced in this case, can lead to worsening back pain, progression of kyphosis, and further vertebral collapse.

The incorrect initial diagnosis of spinal stenosis, combined with limited mobility during quarantine and hospitalization, led to the worsening of back pain due to vertebral collapse and kyphosis. The patient reported being bedbound for the majority of the day during the lockdown and hospitalization and was unable to perform regular stretching exercises. This physical inactivity likely contributed to the weakening of the osteoporotic spine, resulting in further vertebral compression and deformity. An accurate diagnosis of acute vertebral fracture during the initial hospital consultation, followed by appropriate guidance on home exercises and early mobilization, may have mitigated disease progression despite mobility restrictions. This case demonstrates that patients with osteoporosis require close monitoring and management, even during public health crises, to prevent complications from immobility and inactivity.

Early diagnosis and treatment of osteoporotic vertebral fractures are critical for preventing disease progression [17], optimizing function, and reducing morbidity. Adults with osteoporotic vertebral compression fractures should undergo early mobilization, wear a spinal orthosis to reduce pain, exercise, and take calcitonin if their acute pain is analgesic-resistant [18]. The underlying principles and approaches of chiropractic care concentrate on clinical examinations, diagnosis, and management of neuro-muscular-skeletal diseases and their affect on a person's general function [19]. Chiropractor provides therapeutic benefits to scoliosis management [20-23]. A multidisciplinary management plan with pharmacological therapy for osteoporosis, patient education on exercise, and lifestyle modifications can significantly improve pain and quality of life by stabilizing scoliosis, preventing loss of mobility, and mitigating future fracture risk. This case highlights the importance of prompt and appropriate management of fragility fractures during the pandemic to avoid complications, such as rest and inactivity. With telemedicine and home health resources, a continued standard of care can still be provided to this vulnerable population despite mobility restrictions and access challenges.

## Conclusions

During this COVID-19 pandemic with constraints on access and activity, continued vigilance and a standard of care for osteoporosis and vertebral fractures are necessary to avoid complications, such as rest and inactivity. This case highlights the significance of early identification and management of osteoporosis and pathological fractures in the geriatric population. Osteoporosis and pathological vertebral fractures are under-recognized and are often misdiagnosed as osteoarthritis or spinal stenosis. Increased physician awareness and a lower threshold for further workup of back pain in the older population can help identify these conditions early and allow for prompt management and counseling. Astute diagnosis is critical to institute appropriate treatment and prevent disease progression, especially in patients with limited mobility. A comprehensive multidisciplinary approach involving pharmacotherapy, rehabilitation, lifestyle changes, and patient education can significantly improve pain, optimize function, and reduce future fracture risk in these individuals. 
